# Central and peripheral pulse wave velocity and subclinical myocardial stress and damage in older adults

**DOI:** 10.1371/journal.pone.0212892

**Published:** 2019-02-27

**Authors:** Shuiqing Liu, Esther D. Kim, Aozhou Wu, Michelle L. Meyer, Susan Cheng, Ron C. Hoogeveen, Christie M. Ballantyne, Hirofumi Tanaka, Gerardo Heiss, Elizabeth Selvin, Kunihiro Matsushita

**Affiliations:** 1 Department of Epidemiology, Johns Hopkins Bloomberg School of Public Health, Baltimore, Maryland, United States of America; 2 Department of Epidemiology, University of North Carolina Gillings School of Global Public Health, Chapel Hill, North Carolina, United States of America; 3 Department of Medicine, Brigham and Women’s Hospital, Boston, Massachusetts, United States of America; 4 Baylor College of Medicine, Houston, Texas, United States of America; 5 Department of Kinesiology and Health Education, The University of Texas at Austin, Austin, Texas, United States of America; International University of Health and Welfare, School of Medicine, JAPAN

## Abstract

**Background:**

Arterial stiffness independently predicts cardiovascular disease. However, few studies have evaluated the associations of central and peripheral pulse wave velocity (PWV) with biomarkers of both myocardial stress (natriuretic peptide [NT-proBNP]) and damage (high-sensitivity cardiac troponin-T [hs-cTnT]) among persons without cardiac disease.

**Methods:**

We examined 3,348 participants (67–90 years) without prevalent cardiac disease in the Atherosclerosis Risk in Communities (ARIC) Study (2011–13). The cross-sectional associations of PWV quartiles for central arterial segments (carotid-femoral, heart-carotid, heart-femoral) and peripheral artery (femoral-ankle) with NT-proBNP and hs-cTnT were evaluated accounting for potential confounders.

**Results:**

Most PWV measures demonstrated J- or U-shaped associations with the two cardiac biomarkers. The highest (Q4) vs. second lowest (Q2) quartile of central PWV measures (carotid-femoral, heart-carotid, heart-femoral PWV) were associated with higher levels of NT-proBNP independently of demographic characteristics. The associations were less evident for hs-cTnT. These associations were attenuated after adjusting for traditional cardiovascular risk factors, but the heart-carotid PWV-NT-proBNP relationship remained borderline significant (difference in log-NT-proBNP = 0.08 [-0.01, 0.17] in Q4 vs. Q2, p = 0.07). Peripheral PWV demonstrated inverse associations. Higher values of NT-proBNP were seen in the lowest vs. second lowest quartile of all PWV measures.

**Conclusions:**

Central stiffness measures showed stronger associations with cardiac biomarkers (particularly NT-proBNP) than peripheral measures among older adults without cardiac disease. Our findings are consistent with the concept of ventricular-vascular coupling and suggest that central rather than peripheral arterial hemodynamics are more closely related to myocardial stress rather than damage.

## Introduction

Arterial stiffness indicates increased rigidity and decreased elasticity of the arterial wall in response to fluctuations in pulsatile pressure [1] and is considered as an important characteristic of the vascular aging processes [2]. Stiff arteries increase left ventricular (LV) end-systolic pressure and workload, and over time, this can lead to LV hypertrophy (LVH), concentric remodeling [3], and diastolic dysfunction [4]. Indeed, arterial stiffness measures like carotid-femoral pulse wave velocity (cfPWV) are shown to independently predict cardiovascular disease (CVD) [5].

Some studies have demonstrated an association between arterial stiffness and cardiac biomarkers including natriuretic peptides [6–8] and cardiac troponin T (cTnT) [9, 10] among those without clinical cardiac disease, indicating the involvement of arterial stiffness at early stages of the pathogenesis of cardiac disease. However, most studies focused on either cTnT [9, 10] or natriuretic peptide [6–8], investigated clinically selected populations [7, 8, 10], and included small numbers of participants (n<1000) [7, 8, 10]. Furthermore, only a few investigated arterial stiffness in multiple vascular beds [6, 8], and only one study analytically accounted for parameters of cardiac structure and function [7], leaving uncertainty as to whether arterial stiffness independently contributes to cardiac damage or overload.

Therefore, we examined the associations of segment-specific PWV measures with biomarkers of both myocardial stress (natriuretic peptide) and damage (cTnT) in a large cohort of community-dwelling older adults without clinical cardiac disease in the Atherosclerosis Risk in Communities (ARIC) Study.

## Materials and methods

### Study design and population

Data availability and detailed policies for requesting ARIC data can be found at https://www2.cscc.unc.edu/aric/pubs-policies-and-forms-pg. ARIC data can be also obtained from the NHLBI BioLINCC repository (https://biolincc.nhlbi.nih.gov/home/).

ARIC is a community-based cohort study that recruited 15,792 participants aged 45–64 years from Forsyth County, NC, Jackson, MS, suburbs of Minneapolis, MN, and Washington County, MD at baseline (visit 1) during 1987–1989 [11]. The ARIC Study was approved by the institutional review board of each participating center (Wake Forest Baptist Medical Center, Winston-Salem, NC; University of Mississippi Medical Center, Jackson, MS; University of Minnesota, Minneapolis, MN; Johns Hopkins University, Baltimore, MD), and written informed consent was obtained from participants at each visit. Eligible participants for this analysis were 6,538 participants aged 66–90 years who attended visit 5 during 2011–13 when PWV was systematically assessed for the first time in ARIC. We excluded 720 participants with history of coronary heart disease (CHD) (self-reported history at visit 1 or incident cases during follow-up prior to visit 5) or heart failure (prior hospitalization with heart failure or heart failure diagnosis confirmed with the participants’ physicians) (**S1 Fig**).

We further excluded 14 non-white/non-black participants and 673 participants with any missing values of covariates. We also excluded 153 participants without N-terminal pro-B-type natriuretic peptide (NT-proBNP) values and 2 participants without high-sensitivity cTnT (hs-cTnT) values. Finally, we excluded 879 participants without any PWV measures; 494 participants with clinical conditions that impair the quality of the PWV measurement such as body mass index >40 or missing (n = 191), severe arrhythmia like atrial fibrillation at visit 5 (n = 166), self-reported aortic surgery (n = 55), history of peripheral revascularization (n = 25), aortic aneurysm (n = 3), aortic stenosis and aortic regurgitation (n = 48), and LV ejection fraction <30% (n = 6); and 255 participants with any of PWV measures deviating 3 standard deviations from their respective mean. The final analytical sample included 3,348 participants.

### Pulse wave velocity

PWV was defined as the distance between two arterial sites divided by the time the wave transmits that distance, and its higher values indicate greater arterial stiffness [12]. Using an oscillometric device, VP-1000plus (Omron Healthcare, Kyoto, Japan) [13, 14], PWV was measured at the following segments: carotid-femoral (cf), heart-carotid (hc), heart-femoral (hf), and femoral-ankle (fa). The measurement was repeated after 2–5 minutes and the mean PWV was recorded for each segment. For faPWV, the higher value of left and right PWV was used for our primary analysis. cfPWV, hfPWV, and hcPWV were considered to reflect central (elastic) arterial stiffness, faPWV was considered to represent peripheral (muscular) arterial stiffness.

### Cardiac biomarkers

Blood samples were drawn at visit 5 and laboratory tests were performed according to a common protocol by trained technicians at each of the ARIC field centers. NT-proBNP as a biomarker of myocardial stress (or cardiac overload) [15] and hs-cTnT as a biomarker of subclinical myocardial damage [16] were measured on the Roche Elecsys 2010 Analyzer (Roche Diagnostics, Indianapolis, IN 46250) using immunoassay methods [13].

### Covariates of interest

All variables were collected at visit 5 except education level (high school or lower vs. college or above), which was recorded at visit 1. Age, sex/gender, race, current smoking status and current alcohol habit were self-reported. Body mass index was calculated by dividing body weight (kg) by height squared (m^2^). Total cholesterol concentration was determined via an enzymatic method [17]. Sitting blood pressure was measured three times using OMRON HEM -907XL sphygmomanometer (Omron Healthcare, Lake Forest, IL, USA) after a 5-minute rest, and the average of the last two measurements was recorded. Hypertension was defined as systolic blood pressure ≥140 mmHg, diastolic blood pressure ≥90 mmHg, or using antihypertensive medication. Medication use in the past 4 weeks was based on self-report with confirmation of drug containers when possible. Diabetes was defined as hemoglobin A1c ≥6.5%, fasting glucose ≥126 mg/dL, or using diabetic medication or self-reported physician diagnosis of diabetes. Reduced kidney function was defined as creatinine-based estimated glomerular filtration rate (eGFR) <60 mL/min/1.73 m^2^ [18], and urine albumin/creatinine ratio (ACR) ≥30 mg/g was considered as kidney damage [19]. Information of physical activity during leisure time was assessed as a composite score of frequency of TV viewing (“never” as score 5 and “very often” as 1), walking (“never” as score 1 and “very often” as 5), and bicycling (“never” as score 1 and “very often” as 5). LVH was defined as LV mass index >115 g/m^2^ for male and >95 g/m^2^ for female [20]. Concentric remodeling was determined by relative wall thickness >0.42 [20]. Diastolic dysfunction was measured as left atrial volume index (LAVI) ≥34 ml/m^2^ [21]. The cardiac measures were recorded from echocardiogram conducted at visit 5 [22].

### Statistical analyses

Participants’ baseline characteristics were compared across quartiles of each PWV measure using means (± standard deviation [SD]) and ANOVA for normally distributed data, median (interquartile interval [IQI]) and Kruskal-Wallis test for non-normally distributed data, and frequency (percentage) and Pearson’s chi-squared test for categorical data.

To graphically examine the association of each PWV measure with NT-proBNP and hs-cTnT, we visualized the average levels of each cardiac biomarker according to PWV measures adjusting for demographic variables (i.e., age, gender, race, education, and study center) using linear regression models. To allow for potentially non-linear associations, each PWV measure was modeled with its spline terms (knots placed at the thresholds of its quartiles).

Since, indeed, we observed some non-linear relationships (often J- or U-shaped) in several associations, each PWV measure was modeled as quartiles, with the second lowest quartile as a reference. We adjusted for three sets of covariates. Model 1 adjusted for demographic variables (i.e., age, gender, race, education, and study center). Model 2 further adjusted for other cardiovascular risk factors (i.e., body mass index, systolic blood pressure, hypertension medication, smoking status, alcohol habit, physical activity, diabetes, total cholesterol, reduced kidney function, and kidney damage). Model 3 additionally adjusted for the echocardiographic parameters of LV (i.e., LVH, concentric remodeling, and diastolic dysfunction). NT-proBNP and hs-cTnT were log-transformed in linear regression models and dichotomized based on clinical cutpoints (NT-proBNP ≥300 pg/ml [23] and hs-cTnT ≥14 ng/L [24]) in logistic regression models.

We further examined whether the associations with NT-proBNP (based on stronger relationship than hs-cTnT as shown below) were modified by age (≥75 vs. <75 years), gender, race (white vs. black), systolic blood pressure (≥140 vs <140 mmHg), diabetes, smoking status (current vs. former), drinking status (current vs. former), kidney damage, or diastolic dysfunction based on a priori hypothesis by performing stratified analysis adjusting for age, sex, race, and study center. We tested for the interaction between cfPWV (as it is widely considered a standard measure of central arterial stiffness) or hcPWV (based on its consistent positive association with NT-proBNP) and the subgroups using a likelihood ratio test.

All analyses were performed with Stata version 14 (College Station, Texas), and a P-value <0.05 was considered nominally statistically significant.

## Results

### Baseline characteristics

The median age of the 3,348 participants was 74 (IQI 71, 79) years, 39.2% were males, and 77.5% were white (**Table 1**). The median values of NT-proBNP and hs-cTnT were 109 (59, 209) pg/mL and 10 (7, 14) ng/L, respectively. Individuals in the higher quartiles of cfPWV were more likely to be older, black, and more educated, and to have comorbidities including hypertension, diabetes, reduced kidney function, and kidney damage. The prevalence of LVH and concentric remodeling were greater with higher cfPWV. Those with higher values of the other PWV measures were consistently older and had higher systolic blood pressure compared to their counterparts with lower values, but showed varying patterns for other factors (**S1, S2, and S3 Tables**). Specifically, the prevalence of diabetes, reduced kidney function, and kidney damage were positively correlated with central PWV (hcPWV and hfPWV). In contrast, the prevalence of diabetes and reduced kidney function were inversely associated with peripheral PWV (faPWV) but the prevalence of kidney damage was similar across the quartiles of faPWV. The prevalence of LVH and diastolic dysfunction were also inversely correlated with faPWV, whereas at least one of three cardiac echo parameters showed positive relationships to the other PWV measures.

**Table 1:** Baseline characteristics by quartiles of carotid-femoral pulse wave velocity (cfPWV).

Characteristics	cfPWV Q1	cfPWV Q2	cfPWV Q3	cfPWV Q4	Total	P
	(n = 775)	(n = 773)	(n = 769)	(n = 772)	(n = 3,348*)	
Range, cm/s	325–950	951–1124	1125–1324	1325–2248	325–2248	n/a
Age, y	72 (70, 77)	73 (71, 77)	75 (71, 79)	76 (73, 81)	74 (71, 79)	<0.001
Male, %	35.9	38.8	39.3	39.6	39.2	0.41
White, %	81.4	80.5	76.6	68.3	77.5	<0.001
Education, %						
Basic/Intermediate	53.0	46.8	46.6	38.9	46.7	<0.001
Advanced	47.0	53.2	53.4	61.1	53.3	
Study center, %						
Forsyth County, NC	24.0	20.6	19.4	19.2	21.7	<0.001
Jackson, MS	17.2	16.9	22.5	30.3	20.8	
Minneapolis, MN	31.5	35.6	29.1	24.1	30.6	
Washington County, MD	27.4	26.9	29.0	26.4	26.8	
Body mass index, kg/m^2^	27.9 (4.3)	28.2 (4.6)	28.0 (4.4)	27.6 (4.6)	28.0 (4.6)	0.06
Systolic blood pressure, mmHg	123 (15)	128 (16)	133 (16)	138 (18)	131 (17)	<0.001
Diastolic blood pressure, mmHg	65 (9)	67 (10)	67 (10)	68 (10)	67 (10)	<0.001
Antihypertensive drugs, %	60.4	69.3	71.9	76.9	70.0	<0.001
Diabetes, %	24.1	32.0	34.9	43.5	34.0	<0.001
Current smoker, %	6.7	5.3	5.9	5.1	5.8	0.51
Current drinker, %	56.0	54.9	49.8	40.7	50.9	<0.001
Physical activity index, U	2.3 (0.7)	2.4 (0.6)	2.3 (0.6)	2.2 (0.6)	2.3 (0.6)	<0.001
Total cholesterol, mmol/L	4.8 (4.1, 5.6)	4.8 (4.2, 5.5)	4.8 (4.1, 5.5)	4.7 (4.0, 5.4)	4.7 (4.1, 5.5)	0.08
Reduced kidney function, %	20.9	23.3	23.1	30.7	25.1	<0.001
Kidney damage, %	9.5	11.6	17.7	23.6	16.1	<0.001
Left ventricular hypertrophy, %	7.1	7.2	7.4	10.8	8.2	0.02
Left ventricular concentric remodeling, %	38.2	45.3	45.5	51.8	45.5	<0.001
Diastolic dysfunction, %	10.7	9.8	10.4	9.8	10.3	0.92

Values are %, mean (SD), or median (interquartile interval).

* As we kept the maximum number of participants for each PWV in our study, the total number of participants across the quartiles of cfPWV (n = 3,089) does not match the total study population (n = 3,348)

Among the PWV measures, the highest correlation was seen between the two measures reflecting central stiffness, cfPWV and hfPWV (correlation coefficient of 0.841) (**S4 Table**). Overall, hcPWV showed weak correlations with other PWV measures. There was no evident correlation between faPWV and any of three central stiffness measures.

### Continuous relationship between PWV and cardiac markers

**Fig 1:**
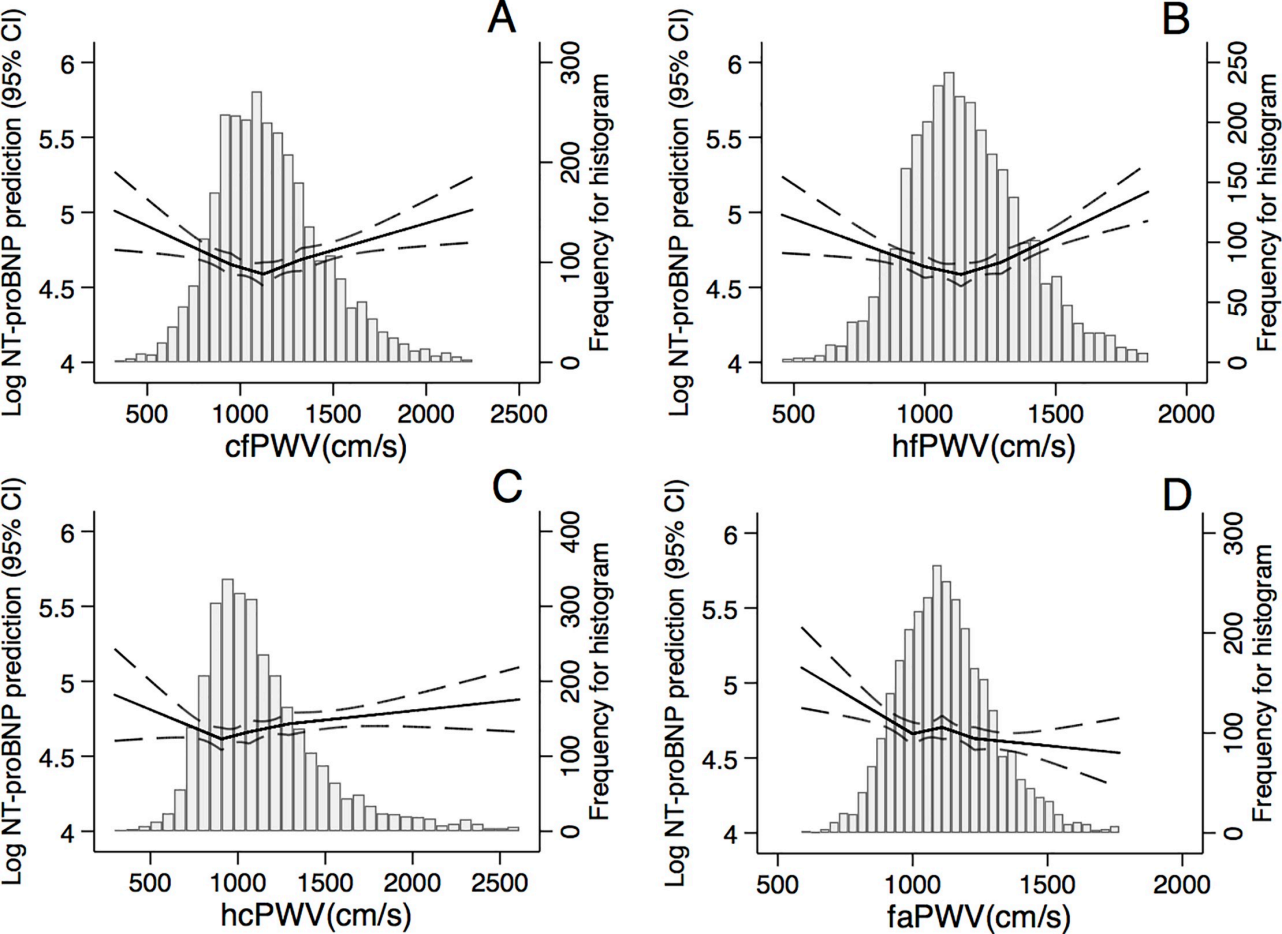
**Associations of central (A-C) and peripheral (D) pulse wave velocity (PWV) measures with NT-proBNP after adjusting for age, sex, race, education, and study center.** (A) cf = carotid-femoral, (B) hf = heart-femoral, (C) hc = heart-carotid, (D) fa = femoral-ankle.

For NT-proBNP, the demographic-adjusted associations with central PWV measures were J- or U-shaped (**Fig 1**). faPWV demonstrated an inverse relationship. In the higher values of PWV, the slope was the steepest for hfPWV (Fig 1B) followed by cfPWV (Fig 1A) and hcPWV (Fig 1C). These patterns were generally consistent in unadjusted models (**S2 Fig**).

**Fig 2:**
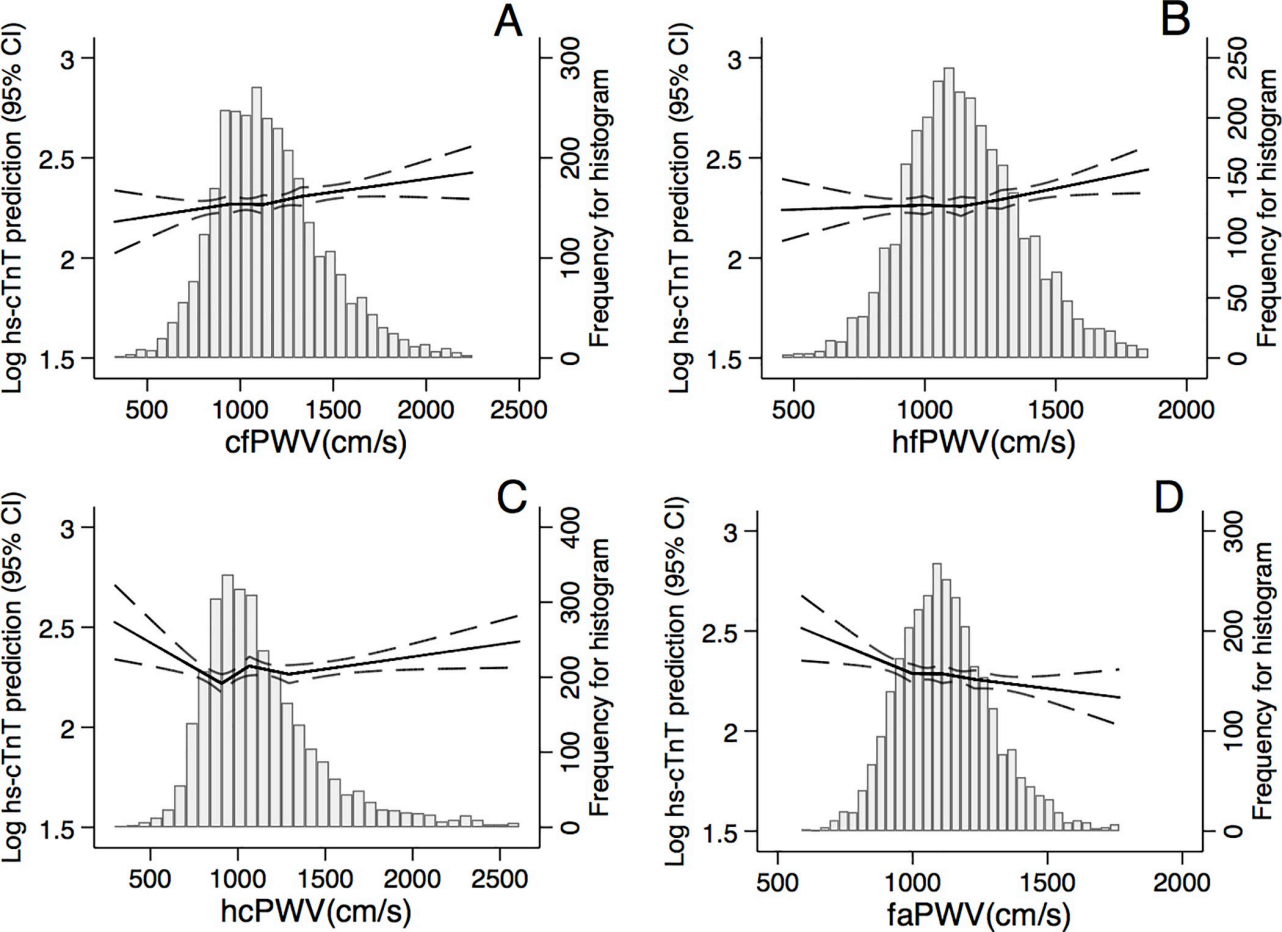
**Associations of central (A-C) and peripheral (D) pulse wave velocity (PWV) measures with hs-cTnT after adjusting for age, sex, race, education, and study center.** (A) cf = carotid-femoral, (B) hf = heart-femoral, (C) hc = heart-carotid, (D) fa = femoral-ankle.

The demographic-adjusted associations of hs-cTnT with PWV were observed to be flatter at each arterial segment (**Fig 2**). faPWV again demonstrated an inverse relationship with hs-cTnT. In unadjusted models, the associations were more evident especially for cfPWV and hfPWV (**S3 Fig**). faPWV was consistently inversely associated with hs-cTnT.

### Quartiles of PWV and NT-proBNP

Adjusting for demographic variables (Model 1), the highest quartile (Q4) of PWV measures reflecting central arterial stiffness were statistically significantly associated with higher NT-proBNP values (**Table 2**). The second highest quartiles (Q3) did not reach statistical significance. Further adjusting for traditional cardiovascular risk factors (Model 2) showed that only the top quartile vs. the second lowest quartile of hcPWV remained borderline significant (Difference in log-NT-proBNP = 0.08 [95%CI: -0.01, 0.17], p = 0.07). Peripheral artery stiffness (faPWV), showed an inverse relationship with NT-proBNP in both Model 1 and Model 2. Significantly higher values of NT-proBNP in the lowest quartile were observed in all PWV measures except in hcPWV in Model 2. Additional adjustment for cardiac echocardiographic parameters slightly attenuated the associations, but general patterns remained similar (Model 3).

**Table 2:** Associations of central and peripheral pulse wave velocity (PWV) measures with NT-proBNP.

		Model 1		Model 2		Model 3	
		Difference in NT-proBNP (95% CI), log-pg/ml	P	Difference in NT-proBNP (95% CI), log-pg/ml	P	Difference in NT-proBNP (95% CI), log-pg/ml	P
**cfPWV** (n = 3089)	**Q1**	0.08 (-0.01, 0.7)	0.09	0.12 (0.03, 0.20)	0.01	0.09 (0.01, 0.17)	0.03
	**Q2**	*ref*		*ref*		*ref*	
	**Q3**	-0.02 (-0.11, 0.07)	0.64	-0.07 (-0.15, 0.02)	0.12	-0.06 (-0.14, 0.02)	0.15
	**Q4**	0.13 (0.04, 0.22)	0.01	0.001 (-0.09, 0.09)	0.99	0.02 (-0.07, 0.11)	0.65
**hfPWV** (n = 3015)	**Q1**	0.13 (0.05, 0.22)	0.003	0.19 (0.10, 0.28)	<0.001	0.15 (0.06, 0.23)	0.001
	**Q2**	*ref*		*ref*		*ref*	
	**Q3**	0.07 (-0.02, 0.16)	0.14	0.01 (-0.08, 0.10)	0.80	-0.01 (-0.09, 0.08)	0.86
	**Q4**	0.19 (0.10, 0.28)	<0.001	0.04 (-0.06, 0.13)	0.45	0.05 (-0.04, 0.13)	0.32
**hcPWV** (n = 3015)	**Q1**	-0.002 (-0.09, 0.09)	0.97	0.01 (-0.07, 0.10)	0.77	-0.001 (-0.09, 0.08)	0.97
	**Q2**	*ref*		*ref*		*ref*	
	**Q3**	0.01 (-0.08, 0.10)	0.80	-0.02 (-0.11, 0.07)	0.65	-0.02 (-0.11, 0.06)	0.59
	**Q4**	0.13 (0.04, 0.22)	0.01	0.08 (-0.01, 0.17)	0.07	0.06 (-0.03, 0.14)	0.17
**faPWV** (n = 3024)	**Q1**	0.09 (0.004, 0.18)	0.04	0.12 (0.04, 0.21)	0.01	0.10 (0.01, 0.18)	0.03
	**Q2**	*ref*		*ref*		*ref*	
	**Q3**	-0.01 (-0.10, 0.08)	0.87	-0.04 (-0.13, 0.05)	0.35	-0.02 (-0.11, 0.06)	0.59
	**Q4**	-0.07 (-0.16, 0.02)	0.12	-0.14 (-0.23, -0.05)	0.002	-0.11 (-0.19, -0.02)	0.02

Model 1 includes the main exposure, age, sex, race, education, study center

Model 2 includes Model 1, body mass index, systolic blood pressure, antihypertensive medication use, smoking, drinking status, diabetes, physical activity index, total cholesterol, reduced kidney function, kidney damage

Model 3 includes Model 2, left ventricular hypertrophy, concentric remodeling, diastolic dysfunction

cf = carotid-femoral, hf = heart-femoral, hc = heart-carotid, fa = femoral-ankle

### Quartiles of PWV and hs-cTnT

Overall the associations of hs-cTnT with PWV measures were less evident (**Table 3**). Specifically, statistically significant associations were observed for the highest quartile (Q4) of cfPWV and hfPWV vs. the reference (Q2) in Model 1, adjusting for demographic factors (Difference in log-hs-cTnT = 0.06 [0.01, 0.12] for cfPWV, 0.07 [0.01, 0.12] for hfPWV). These associations were no longer statistically significant after adjusting for cardiovascular risk factors (Model 2). The highest quartile (Q4) of hcPWV showed positive but weaker and non-significant associations. faPWV was generally inversely correlated with hs-cTnT but statistical significance was only seen in its lowest quartile in Model 1.

**Table 3:** Associations of central and peripheral pulse wave velocity (PWV) measures with hs-cTnT.

		Model 1		Model 2		Model 3	
		Difference in hs-cTnT (95% CI), log-ng/l	P	Difference in hs-cTnT (95% CI), log-ng/l	P	Difference in hs-cTnT (95% CI), log-ng/l	P
**cfPWV** (n = 3089)	**Q1**	-0.01 (-0.07, 0.04)	0.67	0.01 (-0.04, 0.07)	0.59	0.01 (-0.04, 0.06)	0.71
	**Q2**	*ref*		*ref*		*ref*	
	**Q3**	0.03 (-0.02, 0.09)	0.23	0.03 (-0.03, 0.08)	0.35	0.03 (-0.02, 0.08)	0.28
	**Q4**	0.06 (0.01, 0.12)	0.02	0.02 (-0.03, 0.08)	0.45	0.02 (-0.03, 0.08)	0.37
**hfPWV** (n = 3015)	**Q1**	-0.02 (-0.07, 0.04)	0.53	0.003 (-0.05, 0.06)	0.92	-0.01 (-0.06, 0.04)	0.77
	**Q2**	*ref*		*ref*		*ref*	
	**Q3**	-0.002 (-0.06, 0.05)	0.95	-0.01 (-0.07, 0.04)	0.62	-0.02 (-0.07, 0.03)	0.45
	**Q4**	0.07 (0.01, 0.12)	0.02	0.02 (-0.03, 0.08)	0.42	0.02 (-0.03, 0.08)	0.41
**hcPWV** (n = 3015)	**Q1**	-0.02 (-0.08, 0.03)	0.45	-0.01 (-0.07, 0.04)	0.61	-0.02 (-0.07, 0.03)	0.49
	**Q2**	*ref*		*ref*		*ref*	
	**Q3**	0.004 (-0.05, 0.06)	0.90	-0.01 (-0.06, 0.05)	0.81	-0.01 (-0.06, 0.04)	0.76
	**Q4**	0.01 (-0.04, 0.07)	0.70	-0.01 (-0.06, 0.05)	0.79	-0.01 (-0.06, 0.04)	0.65
**faPWV** (n = 3024)	**Q1**	0.06 (0.004, 0.11)	0.04	0.04 (-0.01, 0.10)	0.09	0.03 (-0.02, 0.09)	0.21
	**Q2**	*ref*		*ref*		*ref*	
	**Q3**	-0.02 (-0.07, 0.04)	0.54	-0.01 (-0.06, 0.04)	0.71	-0.01 (-0.06, 0.04)	0.79
	**Q4**	-0.04 (-0.09, 0.02)	0.18	-0.02 (-0.07, 0.03)	0.46	-0.01 (-0.07, 0.04)	0.65

Model 1 includes the main exposure, age, sex, race, education, study center

Model 2 includes Model 1, body mass index, systolic blood pressure, antihypertensive medication use, smoking, drinking status, diabetes, physical activity index, total cholesterol, reduced kidney function, kidney damage

Model 3 includes Model 2, left ventricular hypertrophy, concentric remodeling, diastolic dysfunction

cf = carotid-femoral, hf = heart-femoral, hc = heart-carotid, fa = femoral-ankle

### Quartiles of PWV and clinical elevation of cardiac biomarkers

As anticipated given the limited number of participants above the clinical threshold for each of NT-proBNP (n = 469) and hs-cTnT (n = 958), the results of the logistic regression models were less evident than the linear regression results (**Tables 4 and 5**). Nonetheless, the general patterns were similar, with positive associations between greater central stiffness measures and NT-proBNP in Model 1. Significantly higher odds of NT-proBNP elevation in the lowest vs. second lowest quartile were seen in some PWV measures, particularly in Model 2 (significant for hcPWV and borderline significant for hfPWV).

**Table 4:** Associations of central and peripheral pulse wave velocity (PWV) measures with elevated NT-proBNP (≥300 pg/ml).

		Model 1		Model 2		Model 3	
		OR (95% CI)	P	OR (95% CI)	P	OR (95% CI)	P
**cfPWV** (n = 3089)	**Q1**	1.14 (0.83, 1.56)	0.42	1.28 (0.92, 1.77)	0.15	1.23 (0.88, 1.74)	0.23
	**Q2**	*ref*		*ref*		*ref*	
	**Q3**	0.96 (0.70, 1.32)	0.81	0.87 (0.63, 1.21)	0.40	0.92 (0.65, 1.28)	0.61
	**Q4**	1.35 (0.99, 1.82)	0.05	1.01 (0.74, 1.39)	0.95	1.10 (0.79, 1.54)	0.57
**hfPWV** (n = 3015)	**Q1**	1.17 (0.85, 1.60)	0.33	1.33 (0.96, 1.85)	0.09	1.21 (0.86, 1.69)	0.28
	**Q2**	*ref*		*ref*		*ref*	
	**Q3**	1.00 (0.73, 1.37)	0.99	0.87 (0.62, 1.20)	0.39	0.81 (0.58, 1.14)	0.23
	**Q4**	1.31 (0.96, 1.79)	0.09	0.91 (0.65, 1.27)	0.57	0.94 (0.67, 1.33)	0.74
**hcPWV** (n = 3015)	**Q1**	1.31 (0.96, 1.78)	0.09	1.38 (1.01, 1.90)	0.04	1.33 (0.96, 1.84)	0.09
	**Q2**	*ref*		*ref*		*ref*	
	**Q3**	1.04 (0.76, 1.44)	0.81	0.97 (0.70, 1.35)	0.85	0.95 (0.67, 1.34)	0.76
	**Q4**	1.35 (0.98, 1.84)	0.06	1.20 (0.87, 1.66)	0.27	1.12 (0.80, 1.57)	0.50
**faPWV** (n = 3024)	**Q1**	1.19 (0.87, 1.62)	0.27	1.26 (0.91, 1.74)	0.17	1.14 (0.81, 1.59)	0.46
	**Q2**	*ref*		*ref*		*ref*	
	**Q3**	1.04 (0.77, 1.42)	0.79	0.99 (0.72, 1.37)	0.97	1.06 (0.76, 1.47)	0.75
	**Q4**	0.95 (0.69, 1.30)	0.75	0.83 (0.60, 1.16)	0.27	0.90 (0.64, 1.27)	0.55

OR = odds ratio

Model 1 includes the main exposure, age, sex, race, education, study center

Model 2 includes Model 1, body mass index, systolic blood pressure, antihypertensive medication use, smoking, drinking status, diabetes, physical activity index, total cholesterol, reduced kidney function, kidney damage

Model 3 includes Model 2, left ventricular hypertrophy, concentric remodeling, diastolic dysfunction

cf = carotid-femoral, hf = heart-femoral, hc = heart-carotid, fa = femoral-ankle

**Table 5:** Associations of central and peripheral pulse wave velocity (PWV) measures with elevated hs-cTnT (≥14 ng/l).

		Model 1		Model 2		Model 3	
		OR (95% CI)	P	OR (95% CI)	P	OR (95% CI)	P
**cfPWV** (n = 3089)	**Q1**	1.01 (0.79, 1.31)	0.91	1.10 (0.84, 1.44)	0.49	1.08 (0.82, 1.41)	0.58
	**Q2**	*ref*		*ref*		*ref*	
	**Q3**	1.08 (0.85, 1.39)	0.52	1.07 (0.83, 1.38)	0.62	1.08 (0.84, 1.40)	0.56
	**Q4**	1.36 (1.07, 1.74)	0.01	1.21 (0.94, 1.57)	0.15	1.24 (0.96, 1.61)	0.11
**hfPWV** (n = 3015)	**Q1**	1.02 (0.79, 1.33)	0.86	1.08 (0.82, 1.42)	0.59	1.03 (0.78, 1.35)	0.85
	**Q2**	*ref*		*ref*		*ref*	
	**Q3**	1.03 (0.81, 1.33)	0.79	0.99 (0.76, 1.28)	0.94	0.96 (0.74, 1.25)	0.78
	**Q4**	1.28 (1.00, 1.64)	0.05	1.10 (0.84, 1.43)	0.49	1.10 (0.84, 1.43)	0.50
**hcPWV** (n = 3015)	**Q1**	0.89 (0.68, 1.15)	0.36	0.91 (0.70, 1.20)	0.51	0.90 (0.68, 1.18)	0.43
	**Q2**	*ref*		*ref*		*ref*	
	**Q3**	1.01 (0.79, 1.30)	0.91	0.98 (0.76, 1.27)	0.89	0.98 (0.76, 1.26)	0.85
	**Q4**	1.03 (0.81, 1.32)	0.80	0.96 (0.74, 1.23)	0.73	0.93 (0.72, 1.20)	0.58
**faPWV** (n = 3024)	**Q1**	1.25 (0.98, 1.59)	0.08	1.16 (0.90, 1.50)	0.25	1.11 (0.86, 1.44)	0.42
	**Q2**	*ref*		*ref*		*ref*	
	**Q3**	1.03 (0.81, 1.32)	0.81	1.08 (0.84, 1.40)	0.56	1.09 (0.84, 1.41)	0.51
	**Q4**	0.94 (0.73, 1.21)	0.62	1.00 (0.77, 1.31)	0.97	1.04 (0.80, 1.36)	0.76

OR = odds ratio

Model 1 includes the main exposure, age, sex, race, education, study center

Model 2 includes Model 1, body mass index, systolic blood pressure, antihypertensive medication use, smoking, drinking status, diabetes, physical activity index, total cholesterol, reduced kidney function, kidney damage

Model 3 includes Model 2, left ventricular hypertrophy, concentric remodeling, diastolic dysfunction

cf = carotid-femoral, hf = heart-femoral, hc = heart-carotid, fa = femoral-ankle

Again, overall less evident associations were seen for hs-cTnT (**Table 5**). Higher odds of hs-cTnT elevation were seen in several PWV parameters but statistical significance was only seen in the highest quartile of cfPWV in Model 1.

### Subgroup analysis

There was no statistically significant effect modification in the association between cfPWV and NT-proBNP (based on Model 1) by age, sex, race, high systolic blood pressure, diabetes, smoking status, drinking status, kidney damage, or diastolic dysfunction (**S5 Table**). The association between hcPWV and NT-proBNP was also consistent within categories of the subgroups, without significant interactions (**S6 Table**).

## Discussion

Among community-dwelling older adults without cardiac disease, higher values of central PWV measures (cfPWV, hcPWV, and hfPWV) were associated with higher levels of NT-proBNP, independently of demographic characteristics. Although these associations were considerably attenuated once adjusting for traditional cardiovascular risk factors, the relationship between the highest hcPWV quartile and higher levels of NT-proBNP remained borderline significant. faPWV, which is representative of peripheral stiffness, was generally inversely associated with both cardiac biomarkers. Of interest, higher values of NT-proBNP in the lowest quartile than in the second lowest quartile were seen for most PWV measures. Overall, the associations were weaker for hs-cTnT than for NT-proBNP.

The present study is one of the first to comprehensively examine central and peripheral PWV measures with both NT-proBNP and hs-cTnT in older adults without prevalent cardiac disease. Our main findings of a positive association between measures of central arterial stiffness and cardiac biomarkers are largely consistent with previous studies [7, 8]; however, the associations observed in our study were weaker overall. Although measurement issues may play a role when associations are weaker than expected, this seems unlikely in the ARIC Study, as PWV was measured by trained and certified technicians using a standardized semi-automated protocol with acceptable repeatability [25]. Our study population, exclusively consisting of older whites and blacks (mean age 74 years, range 67–90 years), may play some role in the weaker association. As aging is a prominent risk factor for arterial stiffness [26], it is possible that the variation of PWV measures may not have been large enough to be associated with cardiac biomarkers within this specific population. Nonetheless, further investigations specifically in older adults are warranted, as a Chinese study with ~1,500 individuals reported opposite patterns (stronger association between cfPWV and troponin levels in older [≥60 years] vs. younger [<60 years] individuals) [9].

We found that the overall associations between central PWV measures (cfPWV, hfPWV, and hcPWV) with NT-proBNP or hs-cTnT were stronger than peripheral arterial stiffness (faPWV). This result is consistent with previous studies that investigating similar associations [6, 8, 9], and highlights the pathophysiological importance of central (elastic) arterial stiffness over peripheral (muscular) arterial stiffness. This finding is intuitive since central arteries are anatomically close to the heart and their elasticity is key for effective buffering and cushioning of cardiac pulsations (i.e., ventricular-vascular coupling) [27]. Thus, their abnormal changes may impact the heart more than that of conduit arteries such as femoral and popliteal arteries, which are stiffer and contain more collagen than central arteries [28, 29].

The inverse associations between faPWV, a measure of peripheral stiffness, and both cardiac biomarkers are of interest. Although this is the first study, to our knowledge, to demonstrate this inverse relationship, two studies similarly reported an inverse association for carotid-radial PWV (another measure of peripheral stiffness) among adults without CVD [6, 8]. The underlying pathophysiological mechanism is unclear, but may be related to the fact that PWV of the lower-limb arteries might be lower with significant leg artery stenosis [30]. More specifically, some individuals in the lowest category of PWV may have peripheral artery disease, which is prevalent in older adults [31] and associated with heart failure [32]. To account for this possibility, we excluded participants with ankle-brachial index (ABI) ≤0.9, however, the inverse association persisted (**S7 Table**). Residual confounding may also contribute to the observed inverse association, as some risk factors were differentially associated with faPWV compared to central PWV measures. Nonetheless, our multivariable models adjusted for a wide range of potential confounders.

In our study, the associations with PWV measures were more evident for NT-proBNP than for hs-cTnT, which is largely consistent with the only previous study assessing both NT-proBNP and hs-cTnT [33]. This observation is consistent with the concept of ventricular-vascular coupling as a key element behind the development of heart failure, as NT-proBNP is known to reflect volume overload and ventricular wall stress [34]. On the other hand, the actual mechanisms leading to the release of hs-cTnT to systemic circulation in persons without acute coronary syndrome are not well understood [35]. Although future studies are warranted to confirm, our study suggests that neither central nor peripheral arterial stiffness may play a pivotal role in the subclinical elevation of cTnT in older adults.

We found higher values of NT-proBNP in the lowest quartile than in the second lowest quartile for most PWV measures, resulting in overall J- or U-shaped associations. Although the reasons for such associations are unclear and previous studies did not report a similar pattern, a J-shaped association between clinical characteristics and CVD has been shown for various factors, like blood pressure [36], blood glucose [37], and adiposity [38]. Thus, there may be some individuals in the lowest quartile of PWV with latent high risk of cardiac conditions. Another possibility may be related to the potential beneficial effects of BNP (e.g., vasodilation and glucose utilization) [39], where mildly elevated biological levels of BNP could lead to better artery function. Unfortunately, given our cross-sectional design, we cannot elucidate the temporality of the associations.

Our findings may have important clinical and research implications by suggesting the importance of focusing on arterial stiffness measurements incorporating a central artery component. Furthermore, our results of similar or sometimes stronger relationships of hcPWV and hfPWV over cfPWV suggest the potential usefulness of central arterial stiffness measures other than cfPWV. In particular, hcPWV and hfPWV may have some technical advantage over cfPWV, as cfPWV requires probes on both neck and groin, which can be cumbersome to technicians and the subject [40].

In addition to a cross-sectional design described above, there are a few limitations in our study. As with any observational study, we cannot rule out the possibility of residual confounding although we included several important confounders in our models. Also, as our study population includes mainly older white participants and most black participants were from Jackson, MS, generalization of our findings to younger population or other ethnic groups should be done carefully. Moreover, this study population may seem highly selected with participants who have survived and are healthier than those who died. However, given high retention rate in ARIC over 30 years, it seems likely that our study population is less selected as compared to a scenario of establishing a cohort of older adults de novo.

In conclusion, among older adults without prevalent cardiac disease, central PWV measures were associated with higher levels of NT-proBNP but less so with hs-cTnT. These findings are consistent with the concept of ventricular-vascular coupling, whereby central rather than peripheral arterial hemodynamics are more directly related to myocardial stress rather than damage. Our study further supports the pathophysiological importance of central arterial stiffness over peripheral arterial stiffness in subclinical cardiac stress.

## Supporting information

S1 Fig Study flow diagram. (PDF)

S2 Fig Unadjusted associations of central (A-C) and peripheral (D) pulse wave velocity (PWV) measures with NT-proBNP.
(PDF)

S3 Fig Unadjusted associations of central (A-C) and peripheral (D) pulse wave velocity (PWV) measures with hs-cTnT.
(PDF)

S1 Table Baseline characteristics by quartiles of heart-femoral pulse wave velocity (hfPWV). (PDF)

S2 Table Baseline characteristics by quartiles of heart-carotid pulse wave velocity (hcPWV). (PDF)

S3 Table Baseline characteristics by quartiles of femoral-ankle pulse wave velocity (faPWV). (PDF)

S4 Table Pearson correlation coefficients among pulse wave velocity (PWV) measures. (PDF)

S5 Table Subgroup and interaction analysis of the association between carotid-femoral pulse wave velocity and NT-proBNP. (PDF)

S6 Table Subgroup and interaction analysis of the association between heart-carotid pulse wave velocity and NT-proBNP. (PDF)

S7 Table Associations of femoral-ankle pulse wave velocity (faPWV) with NT-proBNP after excluding participants with ankle-brachial index ≤0.9. (PDF)

## Abbreviations

ACR: albumin/creatinine ratio
ARIC Study: Atherosclerosis Risk in Communities Study
cfPWV: carotid-femoral pulse wave velocity
CHD: coronary heart disease
CVD: cardiovascular disease
eGFR: estimated glomerular filtration rate
faPWV: femoral-ankle pulse wave velocity
hcPWV: heart-carotid pulse wave velocity
hfPWV: heart-femoral pulse wave velocity
hs-cTnT: high-sensitivity cardiac troponin-T
IQI: interquartile interval
LAVI: left atrial volume index
LVH: left ventricular hypertrophy
NT-proBNP: N-terminal pro-B-type natriuretic peptide
OR: odds ratio
PWV: pulse wave velocity
SD: standard deviation
