# Supplementary figures and images for: Central and peripheral pulse wave velocity and subclinical myocardial stress and damage in older adults
Source: PLoS One. 2019 Feb 27;14(2):e0212892. doi: 10.1371/journal.pone.0212892 (PMC6392306; doi:10.1371/journal.pone.0212892)

**S1 Fig: Study flow diagram**

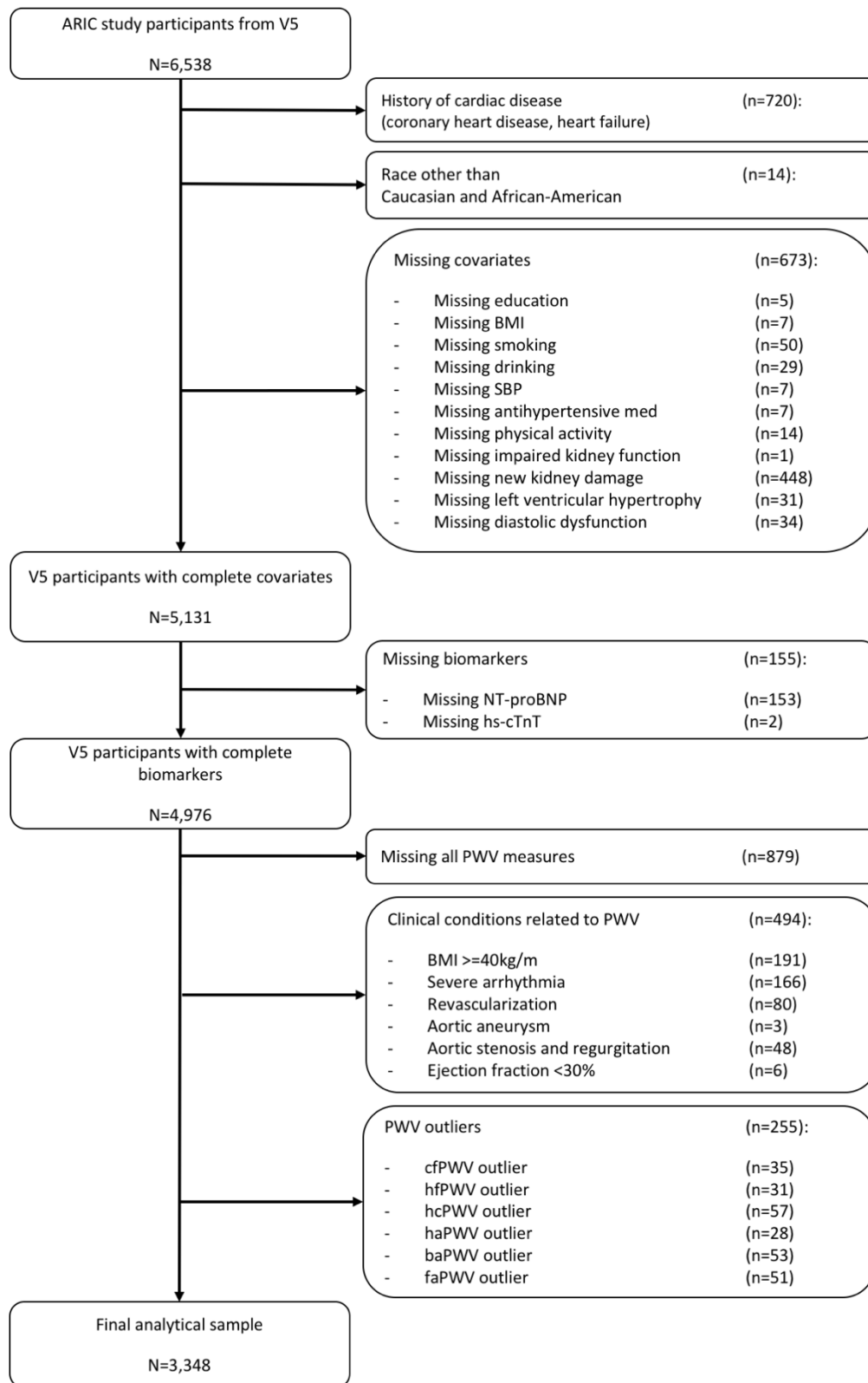

Supplement: S1 Fig — (PDF) [file pone.0212892.s001.pdf]
