# Supplementary material for: Central and peripheral pulse wave velocity and subclinical myocardial stress and damage in older adults
Source: PLoS One. 2019 Feb 27;14(2):e0212892. doi: 10.1371/journal.pone.0212892 (PMC6392306; doi:10.1371/journal.pone.0212892)
Supplement: S2 Fig — Unadjusted associations of central (A-C) and peripheral (D) pulse wave velocity (PWV) measures with NT-proBNP. (PDF) [file pone.0212892.s002.pdf]

S2 Fig: Unadjusted associations of central (A-C) and peripheral (D) pulse wave velocity (PWV) measures with NT-proBNP

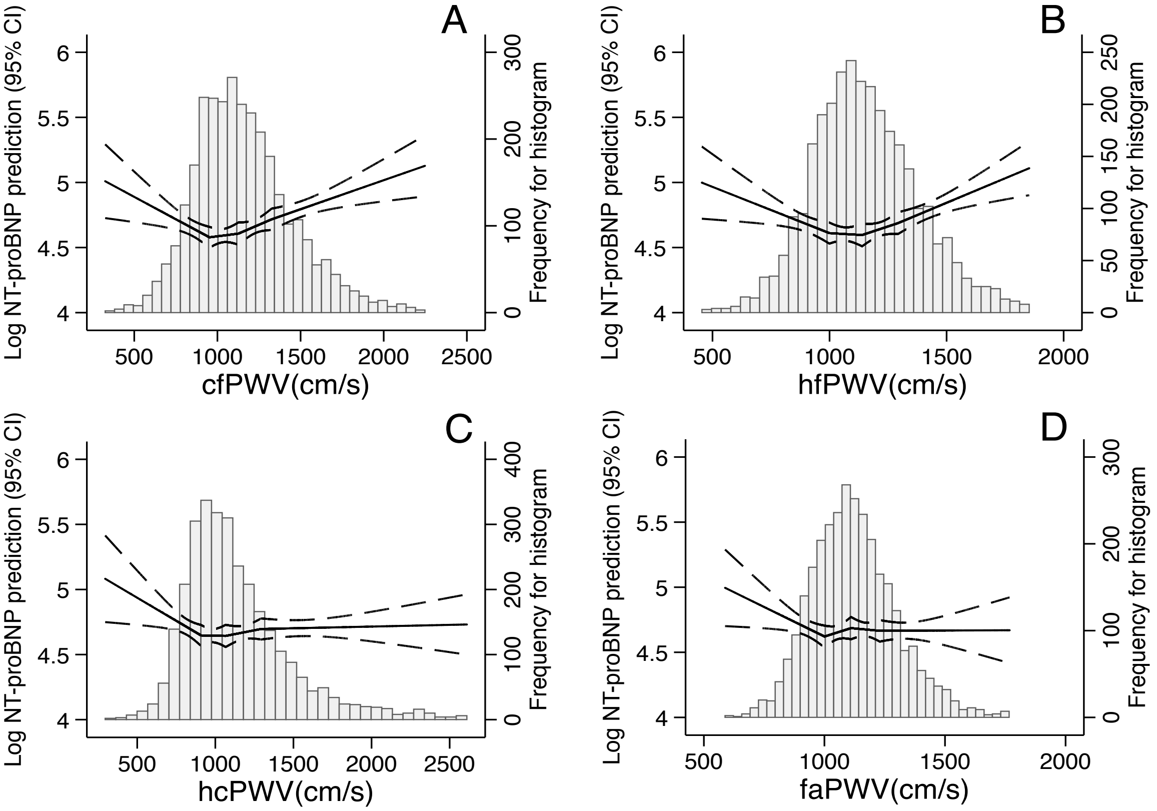

(A) cf=carotid-femoral, (B) hf=heart-femoral, (C) hc=heart-carotid, (D) fa=femoral-ankle
