# Supplementary material for: Central and peripheral pulse wave velocity and subclinical myocardial stress and damage in older adults
Source: PLoS One. 2019 Feb 27;14(2):e0212892. doi: 10.1371/journal.pone.0212892 (PMC6392306; doi:10.1371/journal.pone.0212892)
Supplement: S1 Table — (PDF) [file pone.0212892.s004.pdf]

S1 Table: Baseline characteristics by quartiles of heart-femoral pulse wave velocity (hfPWV)

Characteristics	**hfPWV** Q1 (n=756)	**hfPWV** Q2 (n=757)	**hfPWV** Q3 (n=753)	**hfPWV** Q4 (n=749)	**Total** (n=3,348*)
Range, cm/s	455-998	999-1138	1139-1291	1292-1853	455-1853
Age, y	72 (70, 76)	73 (71, 78)	75 (72, 79)	76 (73, 81)	74 (71, 79)
Male, %	28.7	33.8	39.8	50.1	39.2
White, %	81.7	81.2	76.4	67.7	77.5
Education, %					
Basic/Intermediate	46.4	49.0	45.2	44.7	46.7
Advanced	53.6	51.0	54.8	55.3	53.3
Study center, %					
Forsyth County, NC	20.1	24.2	19.9	18.4	21.7
Jackson, MS	17.2	16.9	22.3	30.3	20.8
Minneapolis, MN	29.6	30.5	30.9	30.0	30.6
Washington County, MD	33.1	28.4	26.8	21.2	26.8
Body mass index, kg/m^2	28.5 (4.4)	28.0 (4.7)	27.9 (4.5)	27.3 (4.3)	28.0 (4.6)
Systolic blood pressure, mmHg	123 (16)	128 (15)	133 (16)	139 (18)	131 (17)
Diastolic blood pressure, mmHg	65 (10)	66 (10)	68 (10)	69 (10)	67 (10)
Antihypertensive drugs, %	63.5	68.6	70.9	74.5	70.0
Diabetes, %	28.0	31.8	33.6	40.7	34.0
Current smoker, %	6.9	4.9	4.9	5.5	5.8
Current drinker, %	54.1	51.8	50.3	45.0	50.9
Physical activity index, U	2.3 (0.7)	2.3 (0.6)	2.3 (0.7)	2.2 (0.6)	2.3 (0.6)
Total cholesterol, mmol/L	4.8 (4.2, 5.6)	4.8 (4.2, 5.6)	4.8 (4.1, 5.5)	4.6 (4.0, 5.5)	4.7 (4.1, 5.5)
Reduced kidney function, %	20.4	22.5	24.2	29.5	25.1
Kidney damage, %	9.0	12.3	16.3	23.8	16.1
Left ventricular hypertrophy, %	8.3	7.0	8.4	8.9	8.2
Left ventricular concentric remodeling, %	39.2	44.5	46.2	50.5	45.5
Diastolic dysfunction, %	11.1	7.3	12.1	9.7	10.3

Values are %, mean (SD), or median (interquartile interval).
* As we kept the maximum number of participants for each PWV in our study, the total number of participants across the quartiles of hfPWV (n=3,015) does not match the total study population (n=3,348)
