# Supplementary material for: Central and peripheral pulse wave velocity and subclinical myocardial stress and damage in older adults
Source: PLoS One. 2019 Feb 27;14(2):e0212892. doi: 10.1371/journal.pone.0212892 (PMC6392306; doi:10.1371/journal.pone.0212892)
Supplement: S2 Table — (PDF) [file pone.0212892.s005.pdf]

S2 Table: Baseline characteristics by quartiles of heart-carotid pulse wave velocity (hcPWV)

**Characteristics**	**hcPWV** Q1 (n=754)	**hcPWV** Q2 (n=756)	**hcPWV** Q3 (n=752)	**hcPWV** Q4 (n=753)	**Total** (n=3,348*)
Range, cm/s	295-906	907-1064	1065-1291	1292-2614	295-2614
Age, y	74 (71, 78)	74 (71, 78)	74 (71, 79)	75 (72, 79)	74 (71, 79)
Male, %	19.1	32.0	44.4	56.8	39.2
White, %	78.0	78.2	73.9	77.0	77.5
Education, %					
Basic/Intermediate	41.6	47.9	45.7	50.1	46.7
Advanced	58.4	52.1	54.3	49.9	53.3
Study center, %					
Forsyth County, NC	18.4	24.2	21.1	18.9	21.7
Jackson, MS	21.5	20.1	23.7	21.4	20.8
Minneapolis, MN	32.0	30.6	26.6	32.0	30.6
Washington County, MD	28.1	25.1	28.6	27.8	26.8
Body mass index, kg/m^2	27.9 (4.8)	28.0 (4.6)	27.9 (4.3)	27.9 (4.2)	28.0 (4.6)
Systolic blood pressure, mmHg	128 (17)	130 (16)	132 (18)	133 (17)	131 (17)
Diastolic blood pressure, mmHg	66 (10)	67 (10)	67 (10)	67 (10)	67 (10)
Antihypertensive drugs, %	68.8	69.8	69.7	69.1	70.0
Diabetes, %	32.1	32.4	35.1	34.5	34.0
Current smoker, %	6.2	5.8	4.8	5.3	5.8
Current drinker, %	48.5	48.7	50.3	53.8	50.9
Physical activity index, U	2.3 (0.6)	2.3 (0.7)	2.3 (0.6)	2.3 (0.6)	2.3 (0.6)
Total cholesterol, mmol/L	4.9 (4.2, 5.6)	4.8 (4.1, 5.6)	4.7 (4.1, 5.5)	4.6 (4.0, 5.4)	4.7 (4.1, 5.5)
Reduced kidney function, %	22.8	22.0	25.4	26.3	25.1
Kidney damage, %	11.4	13.9	16.5	19.5	16.1
Left ventricular hypertrophy, %	9.5	6.6	8.5	8.0	8.2
Left ventricular concentric remodeling, %	45.4	46.2	46.5	42.2	45.5
Diastolic dysfunction, %	9.3	9.1	9.3	12.5	10.3

Values are %, mean (SD), or median (interquartile interval).
* As we kept the maximum number of participants for each PWV in our study, the total number of participants across the quartiles of hcPWV (n=3,015) does not match the total study population (n=3,348)
