# Supplementary material for: Central and peripheral pulse wave velocity and subclinical myocardial stress and damage in older adults
Source: PLoS One. 2019 Feb 27;14(2):e0212892. doi: 10.1371/journal.pone.0212892 (PMC6392306; doi:10.1371/journal.pone.0212892)
Supplement: S3 Table — (PDF) [file pone.0212892.s006.pdf]

S3 Table: Baseline characteristics by quartiles of femoral-ankle pulse wave velocity (faPWV)

Characteristics	**faPWV** Q1 (n=756)	**faPWV** Q2 (n=758)	**faPWV** Q3 (n=754)	**faPWV** Q4 (n=756)	**Total** (n=3,348*)
Range, cm/s	588-999	1000-1109	1110-1228	1229-1766	588-1766
Age, y	74 (71, 78)	74 (71, 78)	74 (71, 79)	75 (71, 79)	74 (71, 79)
Male, %	38.5	38.8	38.9	38.1	39.2
White, %	67.5	75.6	79.4	84.1	77.5
Education, %					
Basic/Intermediate	45.0	49.1	48.0	43.7	46.7
Advanced	55.0	50.9	52.0	56.3	53.3
Study center, %					
Forsyth County, NC	14.9	16.8	22.1	29.5	21.7
Jackson, MS	30.8	22.8	19.2	14.4	20.8
Minneapolis, MN	26.1	33.6	32.9	27.4	30.6
Washington County, MD	28.2	26.8	25.7	28.7	26.8
Body mass index, kg/m^2	29.3 (4.7)	28.1 (4.4)	27.5 (4.3)	26.8 (4.2)	28.0 (4.6)
Systolic blood pressure, mmHg	126 (17)	129 (17)	132 (17)	135 (18)	131 (17)
Diastolic blood pressure, mmHg	63 (9)	65 (9)	68 (10)	70 (10)	67 (10)
Antihypertensive drugs, %	76.2	69.9	68.2	63.6	70.0
Diabetes, %	37.0	35.6	31.6	30.4	34.0
Current smoker, %	8.1	5.1	4.4	5.0	5.8
Current drinker, %	43.4	52.8	52.9	52.4	50.9
Physical activity index, U	2.2 (0.6)	2.3 (0.7)	2.3 (0.6)	2.3 (0.6)	2.3 (0.6)
Total cholesterol, mmol/L	4.7 (4.1, 5.5)	4.7 (4.0, 5.5)	4.8 (4.1, 5.5)	4.9 (4.2, 5.6)	4.7 (4.1, 5.5)
Reduced kidney function, %	29.6	25.1	22.4	21.2	25.1
Kidney damage, %	15.5	14.2	16.4	15.9	16.1
Left ventricular hypertrophy, %	11.0	8.0	6.8	6.7	8.2
Left ventricular concentric remodeling, %	45.5	41.4	46.2	47.1	45.5
Diastolic dysfunction, %	13.8	9.9	9.5	7.5	10.3

Values are %, mean (SD), or median (interquartile interval).
* As we kept the maximum number of participants for each PWV in our study, the total number of participants across the quartiles of faPWV (n=3,024) does not match the total study population (n=3,348)
