# Supplementary material for: Central and peripheral pulse wave velocity and subclinical myocardial stress and damage in older adults
Source: PLoS One. 2019 Feb 27;14(2):e0212892. doi: 10.1371/journal.pone.0212892 (PMC6392306; doi:10.1371/journal.pone.0212892)
Supplement: S4 Table — (PDF) [file pone.0212892.s007.pdf]

S4 Table: Pearson correlation coefficients among pulse wave velocity (PWV) measures

	**cfPWV**	**hfPWV**	**hcPWV**	**faPWV**
**cfPWV**	1.000			
**hfPWV**	0.841	1.000		
**hcPWV**	-0.002	0.429	1.000	
**faPWV**	-0.014	-0.001	0.041	1.000

cf=carotid-femoral, hf=heart-femoral, hc=heart-carotid, ha=heart-ankle, ba=brachial-ankle, fa=femoral-ankle
