# Supplementary material for: Central and peripheral pulse wave velocity and subclinical myocardial stress and damage in older adults
Source: PLoS One. 2019 Feb 27;14(2):e0212892. doi: 10.1371/journal.pone.0212892 (PMC6392306; doi:10.1371/journal.pone.0212892)
Supplement: S5 Table — (PDF) [file pone.0212892.s008.pdf]

S5 Table: Subgroup and interaction analysis of the association between carotid-femoral pulse wave velocity and NT-proBNP

	Δ NT-proBNP (95% CI), log-pg/ml				P for interaction
	**Q1**	**Q2**	**Q3**	**Q4**	
**Age**					
**<75 years**	0.10 (-0.01, 0.22)	*ref*	0.01 (-0.11, 0.13)	0.20 (0.07, 0.34)	0.29
**>=75 years**	0.06 (-0.08, 0.20)	*ref*	-0.06 (-0.18, 0.07)	0.05 (-0.07, 0.18)	
**Sex**					
**Male**	0.08 (-0.07, 0.24)	*ref*	-0.03 (-0.19, 0.12)	0.18 (0.03, 0.34)	0.20
**Female**	0.06 (-0.05, 0.17)	*ref*	-0.01 (-0.12, 0.10)	0.09 (-0.02, 0.20)	
**Race**					
**White**	0.06 (-0.03, 0.16)	*ref*	-0.03 (-0.13, 0.06)	0.10 (-0.001, 0.20)	0.74
**Black**	0.14 (-0.08, 0.37)	*ref*	0.05 (-0.17, 0.27)	0.23 (0.02, 0.43)	
**Systolic blood pressure**					
**<140**	0.10 (0.004, 0.20)	*ref*	-0.06 (-0.16, 0.05)	0.05 (-0.06, 0.16)	0.99
**>=140**	0.12 (-0.09, 0.33)	*ref*	-0.03 (-0.19, 0.14)	0.09 (-0.07, 0.25)	
**Diabetes**					
**No**	0.08 (-0.03, 0.18)	*ref*	0.004 (-0.10, 0.11)	0.20 (0.09, 0.31)	0.20
**Yes**	0.07 (-0.11, 0.24)	*ref*	-0.08 (-0.24, 0.08)	0.03 (-0.12, 0.19)	
**Current smoker**					
**No**	0.08 (-0.01, 0.18)	*ref*	-0.03 (-0.12, 0.06)	0.12 (0.03, 0.22)	0.48
**Yes**	-0.07 (-0.46, 0.32)	*ref*	0.06 (-0.35, 0.46)	0.22 (-0.21, 0.64)	
**Current drinker**					
**No**	0.12 (-0.01, 0.26)	*ref*	-0.02 (-0.16, 0.11)	0.13 (-0.0001, 0.26)	0.65
**Yes**	0.04 (-0.07, 0.16)	*ref*	-0.27 (-0.14, 0.10)	0.13 (0.001, 0.26)	
**Kidney damage**					
**No**	0.09 (-0.004, 0.18)	*ref*	-0.01 (-0.10, 0.09)	0.10 (-0.002, 0.19)	0.45
**Yes**	0.03 (-0.26, 0.32)	*ref*	-0.15 (-0.40, 0.11)	0.13 (-0.11, 0.38)	
**Diastolic dysfunction**					
**No**	0.05 (-0.05, 0.13)	*ref*	-0.01 (-0.10, 0.08)	0.12 (0.03, 0.21)	0.08
**Yes**	0.28 (-0.02, 0.57)	*ref*	-0.06 (-0.36, 0.24)	0.37 (0.06, 0.68)	

Models adjusted for age, sex, race, and study center
