# Supplementary material for: Central and peripheral pulse wave velocity and subclinical myocardial stress and damage in older adults
Source: PLoS One. 2019 Feb 27;14(2):e0212892. doi: 10.1371/journal.pone.0212892 (PMC6392306; doi:10.1371/journal.pone.0212892)
Supplement: S6 Table — (PDF) [file pone.0212892.s009.pdf]

S6 Table: Subgroup and interaction analysis of the association between heart-carotid pulse wave velocity and NT-proBNP

	Δ NT-proBNP (95% CI), log-pg/ml				P for interaction
	Q1	Q2	Q3	Q4	
**Age**					
**<75 years**	-0.10 (-0.22, 0.02)	*ref*	-0.05 (-0.17, 0.08)	0.13 (0.003, 0.26)	0.09
**>=75 years**	0.11 (-0.02, 0.24)	*ref*	0.07 (-0.05, 0.20)	0.12 (-0.003, 0.25)	
**Sex**					
**Male**	-0.02 (-0.21, 0.18)	*ref*	0.01 (-0.15, 0.17)	0.08 (-0.07, 0.23)	0.75
**Female**	-0.003 (-0.10, 0.10)	*ref*	0.01 (-0.10, 0.12)	0.18 (0.06, 0.29)	
**Race**					
**White**	-0.02 (-0.12, 0.08)	*ref*	0.001 (-0.10, 0.10)	0.09 (-0.004, 0.19)	0.22
**Black**	0.05 (-0.16, 0.27)	*ref*	0.06 (-0.15, 0.26)	0.27 (0.06, 0.49)	
**Systolic blood pressure**					
**<140**	0.01 (-0.09, 0.12)	*ref*	0.01 (-0.09, 0.12)	0.11 (0.004, 0.22)	0.96
**>=140**	-0.02 (-0.19, 0.16)	*ref*	-0.05 (-0.21, 0.12)	0.10 (-0.07, 0.26)	
**Diabetes**					
**No**	0.03 (-0.07, 0.14)	*ref*	0.01 (-0.10, 0.12)	0.12 (0.01, 0.22)	0.17
**Yes**	-0.08 (-0.24, 0.09)	*ref*	0.03 (-0.13, 0.19)	0.18 (0.02, 0.34)	
**Current smoker**					
**No**	0.02 (-0.07, 0.11)	*ref*	0.02 (-0.07, 0.11)	0.13 (0.04, 0.23)	0.21
**Yes**	-0.36 (-0.76, 0.03)	*ref*	-0.07 (-0.50, 0.37)	0.07 (-0.35, 0.49)	
**Current drinker**					
**No**	0.10 (-0.03, 0.23)	*ref*	0.03 (-0.10, 0.16)	0.16 (0.03, 0.29)	0.16
**Yes**	-0.11 (-0.23, 0.02)	*ref*	-0.004 (-0.13, 0.12)	0.10 (-0.03, 0.22)	
**Kidney damage**					
**No**	-0.02 (-0.11, 0.08)	*ref*	-0.02 (-0.11, 0.08)	0.08 (-0.02, 0.18)	0.19
**Yes**	0.13 (-0.14, 0.40)	*ref*	0.15 (-0.10, 0.40)	0.31 (0.06, 0.55)	
**Diastolic dysfunction**					
**No**	-0.02 (-0.11, 0.07)	*ref*	0.01 (-0.08, 0.10)	0.10 (0.01, 0.19)	0.69
**Yes**	0.17 (-0.15, 0.50)	*ref*	0.10 (-0.23, 0.43)	0.25 (-0.02, 0.55)	

Models adjusted for age, sex, race, and study center
