# Supplementary material for: Central and peripheral pulse wave velocity and subclinical myocardial stress and damage in older adults
Source: PLoS One. 2019 Feb 27;14(2):e0212892. doi: 10.1371/journal.pone.0212892 (PMC6392306; doi:10.1371/journal.pone.0212892)
Supplement: S7 Table — (PDF) [file pone.0212892.s010.pdf]

S7 Table: Associations of femoral-ankle pulse wave velocity (faPWV) with NT-proBNP after excluding participants with ankle-brachial index ≤0.9

		Model 1 Δ NT-proBNP (95% CI), log-pg/ml	P	Model 2 Δ NT-proBNP (95% CI), log-pg/ml	P	Model 3 Δ NT-proBNP (95% CI), log-pg/ml	P
**Categorical exposure**							
**faPWV** (n=2956)	**Q1**	0.07 (-0.02, 0.17)	0.11	0.11 (0.03, 0.20)	0.01	0.08 (-0.0001, 0.17)	0.05
	**Q2**	*ref*		*ref*		*ref*	
	**Q3**	-0.0001 (-0.09, 0.09)	<0.001	-0.04 (-0.12, 0.05)	0.41	-0.02 (-0.10, 0.06)	0.63
	**Q4**	-0.07 (-0.16, 0.03)	0.16	-0.14 (-0.23, -0.05)	0.002	-0.10 (-0.19, -0.02)	0.02

Model 1 includes the main exposure, age, sex, race, education, study center
Model 2 includes Model 1, body mass index, systolic blood pressure, antihypertensive medication use, smoking, drinking status, diabetes, physical activity index, total cholesterol, reduced kidney function, kidney damage
Model 3 includes Model 2, left ventricular hypertrophy, concentric remodeling, diastolic dysfunction
